# A high throughput approach for the generation of orthogonally interacting protein pairs

**DOI:** 10.1038/s41598-018-19281-6

**Published:** 2018-01-17

**Authors:** Justin Lawrie, Xi Song, Wei Niu, Jiantao Guo

**Affiliations:** 1 Department of Chemistry, University of Nebraska-Lincoln, Lincoln, Nebraska 68588 United States; 2 Department of Chemical & Biomolecular Engineering, University of Nebraska-Lincoln, Lincoln, Nebraska 68588 United States

## Abstract

In contrast to the nearly error-free self-assembly of protein architectures in nature, artificial assembly of protein complexes with pre-defined structure and function *in vitro* is still challenging. To mimic nature’s strategy to construct pre-defined three-dimensional protein architectures, highly specific protein-protein interacting pairs are needed. Here we report an effort to create an orthogonally interacting protein pair from its parental pair using a bacteria-based *in vivo* directed evolution strategy. This high throughput approach features a combination of a negative and a positive selection. The newly developed negative selection from this work was used to remove any protein mutants that retain effective interaction with their parents. The positive selection was used to identify mutant pairs that can engage in effective mutual interaction. By using the cohesin-dockerin protein pair that is responsible for the self-assembly of cellulosome as a model system, we demonstrated that a protein pair that is orthogonal to its parent pair could be readily generated using our strategy. This approach could open new avenues to a wide range of protein-based assembly, such as biocatalysis or nanomaterials, with pre-determined architecture and potentially novel functions and properties.

## Introduction

**Figure 1:**
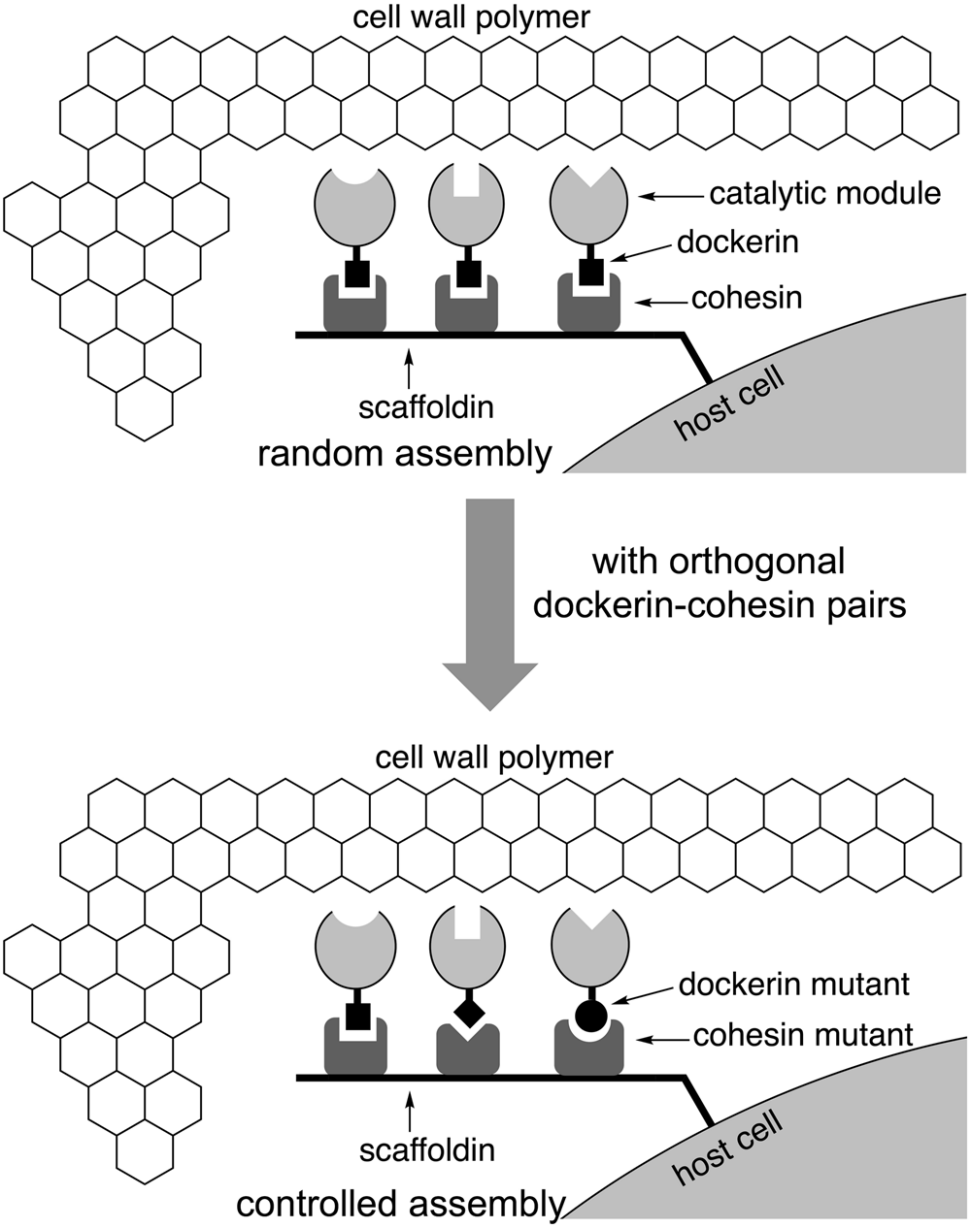
Random and controlled assembly of cellulosome. In nature, the assembly of cellulosomes is mediated by a random attachment of a catalytic module (enzyme), through its dockerin domain, to any cohesin positions on the scaffold protein (scaffoldin). The generation of mutant cohesin-dockerin pairs that are orthogonal to the naturally occurring one may allow a controlled assembly of cellulosomes.

Although significant progress has been made in recent years^1–14^, the precise manipulation of artificial self-assembly of protein complexes *in vitro* remains a great challenge. In contrast, highly ordered permanent or transient protein complexes widely exist in nature and participate in virtually every type of cellular function, including catalysis, structural support, bodily movement, signal transduction, transport, etc. Nature’s error-free self-assembly of protein architectures, such as virus capsids^15^, bacterial carboxysomes^16^, and cellulosomes (Fig. 1)^17–19^ is driven by many weak, noncovalent interactions at protein-protein interfaces^20^. The geometry of subunits in a protein complex is precisely defined by those specific noncovalent interactions^13^. In order to mimic nature’s strategy to construct highly defined three-dimensional protein architectures, we need to have highly specific protein-protein interacting pairs, analogous to G-C and A-T base-pairing interactions in DNA. One potential solution is to explore naturally occurring protein pairs, such as the barnase and barstar pair^21^. The other potential approach is to artificially generate mutually orthogonal protein pairs from a known parent protein pair. This could further expand the repertoire of highly specific protein pairs that are available for the assembly of protein complexes. In addition, such evolved protein pairs are orthogonal but consist of high sequence homology to the parent protein pair and, therefore, have similar physical/chemical properties. This may minimize certain complications when protein pairs with very different properties are used in the assembly of a protein complex.

**Figure 2:**
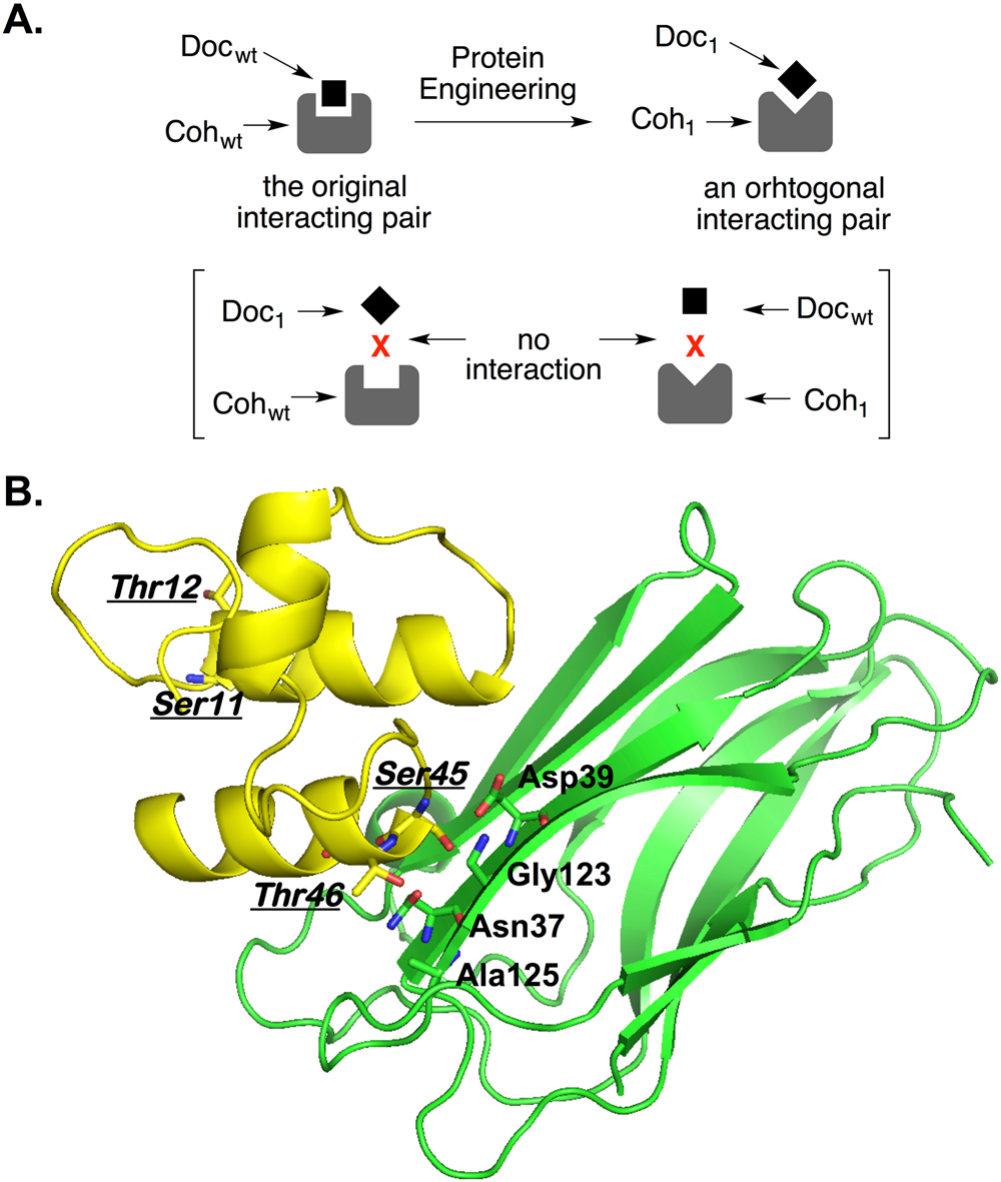
Generation of orthogonal protein pairs from a parent protein pair. (**A**) Generation of mutant dockerin-cohesin (Doc_1_-Coh_1_) pair that is orthogonal to the parental Doc_wt_-Coh_wt_ pair; (**B**) Structures of Doc_wt_-Coh_wt_ pair from *C. thermocellum*. Residues from dockerin (yellow) are labeled in italic and underlined. Residues from cohesin (green) are labeled in regular bold.

To demonstrate the feasibility of the aforementioned approach, we selected a type-I cohesin-dockerin pair from *Clostridium thermocellum* as our model system. High affinity cohesin-dockerin interactions are the basis of self-assembly of cellulosomes^17,18^, which are multi-protein complexes from certain anaerobic bacteria and fungi for a highly efficient degradation of cellulosic material (Fig. 1). A cellulosome consists of a core structural protein (scaffoldin) that serves as a scaffold to connect multiple catalytic enzymes through the interaction between the type I cohesin domains on itself and the type I dockerin domains of catalytic enzymes. Due to the indiscriminatory nature of cohesin-dockerin recognition within a microorganism species, the assembled cellulosomes have diverse molecular composition and structure, which corresponds to heterogeneous catalytic activities for cellulosic material degradation. In this work, we seek to generate a mutant cohesin-dockerin pair (Fig. 2A) that is derived from but orthogonal to the naturally occurring (wild-type) one. The generation of orthogonal cohesin-dockerin pairs will allow controlled assembly of cellulosomes (Fig. 1), which will facilitate current studies of synergistic actions among cellulosomal enzymes^17–19^. The second cohesin domain from the scaffoldin protein (CipA; residues 182–328) and the dockerin domain from a glycoside hydrolase (xylanase 10B; residues 733–791) were used in this study. The crystal structure of the protein complex of these two domains has been reported^22^.

**Figure 3:**
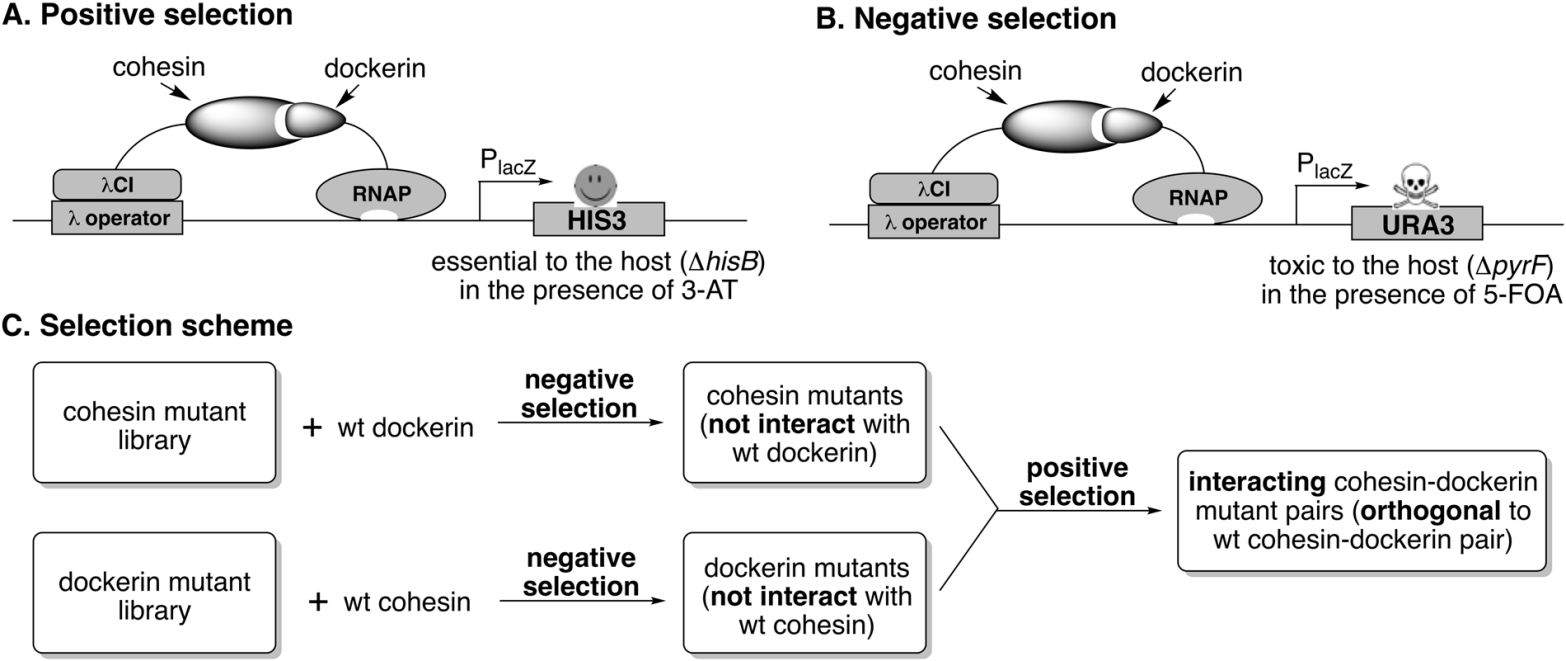
Selection scheme. (**A**) positive selection; (**B**) negative selection; (**C**) selection scheme. The positive selection selects mutant cohesin-dockerin pairs that can engage in effective interaction. The negative selection removes any dockerin or cohesin mutants that retain effective interaction with the parent cohesin or dockerin. The combination of negative and positive selection should yield interacting protein pairs that are orthogonal to their parent. Abbreviations: λcI, bacteriophage λ repressor protein; RNAP, α-subunit of RNA polymerase; P_lacZ_, the lac operon promoter; 3-AT, 3-amino-1,2,4-triazole; 5-FOA, 5-fluoroorotic acid.

A few methods have been developed for a high-throughput engineering of protein-protein interactions. Phage^23^, yeast^24^, and bacterial^25^ displayed protein libraries are generally screened with either panning or fluorescence-activated cell sorting (FACS). In contrast, the yeast two-hybrid system^26^ links protein-protein interactions to a phenotype (e.g., cell growth) that confers a selective advantage to the host, which simplifies the selection process. Bacterial two-hybrid system^27–29^ has also been developed. In comparison to the yeast system, the bacterial system has the advantage of higher transformation efficiency and faster cell growth rate. However, the current bacterial system lacks a negative (counter) selection scheme. In the present study, we devised a bacterial negative selection scheme (Fig. 3B) that is analogous to the yeast one^30,31^. We subsequently demonstrated that an orthogonal mutant pair could be readily obtained through a combination of positive and negative selections (Fig. 3). Throughout the article, we will use Coh_wt_-Doc_wt_ and Coh_1_-Doc_1_ to represent the parent (wild-type) and mutant (evolved) cohesin-dockerin pairs, respectively.

## Results and Discussion

### General approach

In order to generate a mutant cohesin-dockerin pair that is orthogonal to its parent pair, we explored a structure-guided, semi-rational protein engineering approach (Fig. 2). This approach consists of two essential steps: (1) mutagenesis. The important amino acid residues at the protein-protein interface of the parent pair are randomized. Presumably, such modification would completely abolish or significantly weaken the interaction between mutants and their parents; and (2) selection. This process identifies mutant protein pairs that interact to each other, but do not have significant cross interaction with the parent protein pair. Our selection system consists of both a positive selection and a negative selection (Fig. 3). The positive selection selects mutant cohesin-dockerin pairs that can engage in effective interaction (Fig. 3A). The negative selection removes any dockerin or cohesin mutants that retain effective interaction with the parent cohesin or dockerin (Fig. 3B). The combination of negative and positive selection should yield interacting protein pairs that are orthogonal to their parent (Fig. 3C). This selection scheme can likely be generalized and used to create orthogonal pairs for other proteins as well.

### Positive selection system

The BacterioMatch® II two-hybrid system^27–29^ was used as the molecular biology platform for the positive selection (Fig. 3A). Two reasons promoted us to choose bacteria (*E. coli*) two-hybrid over yeast two-hybrid as the positive selection system: (1) *E. coli* grows much faster than yeast; (2) *E. coli* is transformed with higher efficiency so larger libraries can be readily constructed and selected/screened. BacterioMatch II selection is built upon the genetic complementation of the chromosomal *hisB* gene deletion by the episomal expression of the *S. serevisiae HIS3* gene in an *E. coli* host strain. Both genes encode imidazoleglycerol-phosphate dehydratase, which is an essential enzyme in the L-histidine biosynthesis. To study the interaction between a cohesin and a dockerin protein, the cohesin is expressed as a C-terminal fusion protein to the full-length bacteriophage λ repressor protein (λcI), and the dockerin is fused to the N-terminal domain of the α-subunit of RNA polymerase (RNAPα). When both fusion proteins are co-expressed in *E. coli* selection host, if the cohesin and the dockerin variants interact, they recruit and stabilize the binding of RNA polymerase at the promoter and activate the transcription of the *HIS3* reporter gene, which allows cells to grow in the presence of 3-amino-1,2,4-triazole (3-AT), a competitive inhibitor of *HIS3* gene product. In general, a stronger interaction confers the cells resistant to higher concentrations of 3-AT, while lack of interaction only permits cells to survive on media without 3-AT. It should be noted that other factors, such as the protein expression level, may affect the cell growth as well. For example, a protein pair with higher expression level can likely survive higher concentrations of 3-AT than a protein pair with lower expression level. If one would like to compare the interaction strength between two different protein pairs by using the positive selection system, the expression levels of the two pairs need to be adjusted to a similar level (e.g., to manipulate gene transcription level using different concentrations of inducer such as IPTG).

To test if the positive selection works, we examined the interaction between the Coh_wt_-Doc_wt_ pair. To this end, two plasmids were constructed, including pBT-Coh_wt_ (containing the gene that encodes the λcI-Coh_wt_ fusion protein) and pTRG-Doc_wt_ (containing the gene that encodes the RNAPα-Doc_wt_ fusion protein). We initially examined a construct in which Doc_wt_ was directly fused to RNAPα. However, we observed poor cell growth in a two-hybrid study of the Coh_wt_-Doc_wt_ interaction. We hypothesized that such poor cell growth was resulted from the degradation of the RNAPα-Doc_wt_ fusion protein since it was known that dockerin domain is prone to degradation in *Escherichia coli*^22^. According to literature, dockerin-containing enzymes could be expressed as full-length proteins in *E. coli*^32–35^, we decided to improve the stability of the dockerin protein by inserting the X6b carbohydrate-binding domain between RNAPα and the dockerin domain. The X6b domain is naturally fused to the N-terminus of the type I dockerin domain from *C. thermocellum* and does not interact with the cohesin domain^18,19^. The X6b domain was included in all dockerin constructs in this work. As shown in Table 1, cell growth was observed in the presence of 5 mM of 3-AT when both pBT-Coh_wt_ and pTRG-Doc_wt_ were co-transformed into the *E. coli* selection host (entry 1; Table 1). As negative controls, no cell growth was detected when either pBT-Coh_wt_ was co-transformed with the empty pTRG vector or the pTRG-Doc_wt_ was co-transformed with the empty pBT vector (entry 2, 3; Table 1). The results confirmed that cells only grew when both Coh_wt_ and Doc_wt_ proteins were present. In comparison to the positive control provided by the BacterioMatch® II kit, the interaction between Coh_wt_ and Doc_wt_ (entry 1; Table 1) led to similar level of cell growth as the interaction between Gal11P and LGF2 (entry 4; Table 1), which were co-expressed from the pTRG-Gal11P and the pBT-LGF2 positive control plasmids.

**Table 1:** Examination of Coh_wt_ and Doc_wt_ interaction using The BacterioMatch® II two-hybrid system as the positive selection.

entry	plasmids in the selection host	cell growth (cfu^a^)	
		0 mM 3-AT	5 mM 3-AT
1	pBT-Coh_wt_ and pTRG-Doc_wt_	~5 × 10^3^	~1 × 10^3^
2	pBT and pTRG-Doc_wt_	~6 × 10^3^	0
3	pBT-Coh_wt_ and pTRG	~5 × 10^3^	0
4	pTRG-Gal11P and pBT-LGF2	~6 × 10^3^	~1 × 10^3^
5	pBT-Coh_wt_ and pTRG-Doc_AL_	~5 × 10^3^	0

^a^cfu, colony-forming unit.

### Negative selection system

As a critical component to enable the generation of orthogonally interacting protein pairs, we developed a negative selection method (Fig. 2B). We modified the yeast URA3/5-FOA counter selection system^30,31^ into the bacterial two-hybrid system. *URA3* encodes orotidine 5′-phosphate decarboxylase, which catalyzes the transformation of 5-fluoroorotic acid (5-FOA) into a highly toxic compound (5-fluorouracil) and causes cell death. A similar approach was demonstrated in a bacterial one-hybrid system to select for Zn finger proteins^36^.

To enable the selection, we first deleted the *pyrF* gene (encodes orotidine-5′-phosphate decarboxylase) on the chromosome of the BacterioMatch II reporter strain to generate strain WNPPI7. As a result, *E. coli* WNPPI7 lost the ability to convert 5-fluoroorotic acid (5-FOA) into a cytotoxic compound, and therefore survives on solid minimal media containing 5-FOA and uracil supplementation (Entry 3, 4; Table 2). We then modified the F’ plasmid of WNPPI7 to replace the *HIS3* reporter gene with a copy of the *URA3* gene, resulting in strain WNPPI5. However, the 5-FOA tolerance test showed that the basal expression level of URA3 protein in strain WNPPI5 is high enough to lead to cell death of the host strain itself on plates containing 0.5 mM of 5-FOA (Entry 5, 6; Table 2). To solve the problem, we constructed strain WNPPI8, in which the *URA3* gene was inserted behind the *HIS3* reporter gene. As the second gene in an operon, the reduced basal expression level of URA3 in strain WNPPI8 allowed the cells to survive on plates containing 2.5 mM of 5-FOA (Entry 7; Table 2). When an interacting protein pair (LGF2 and Gal11P) was expressed in strain WNPPI8, the increased transcription level of URA3 resulted in cell death in the presence of as low as 0.5 mM of 5-FOA (Entry 8; Table 2). The negative selection system was further evaluated when pBT-Coh_wt_ and pTRG-Doc_wt_ were co-transformed into WNPPI8. 5-FOA concentrations ranging from 0 mM to 2.5 mM were included in the experiment. In comparison to cell growth on plates without 5-FOA, a significant decrease in colony formation unit was observed when the cells were cultured on plates containing 0.2 mM 5-FOA (data not shown). Further increasing the 5-FOA concentration to either 0.5 or 2.5 mM completely eliminated cell growth (Entry 9, Table 2). As controls, no growth defect was observed when WNPPI8 was transformed with either pBT-Coh_wt_ plus pTRG or pTRG-Doc_wt_ plus pBT. The results showed that the URA3/5-FOA negative selection system could efficiently eliminate interacting cohesin and dockerin variants that are generated in the mutagenesis step.

**Table 2:** Examination of negative selection host strains^a^.

	host	protein pair	cell growth (cfu^a^) with or without 5-FOA (mM)		
			0	0.5	2.5
1	BacterioMatch II	none	~1 × 10^3^	0	0
2	BacterioMatch II	Gal11P and LGF2	~1 × 10^3^	0	0
3	WNPPI7	none	~1 × 10^3^	~1 × 10^3^	~1 × 10^3^
4	WNPPI7	Gal11P and LGF2	~1 × 10^3^	~1 × 10^3^	~1 × 10^3^
5	WNPPI5	none	~1 × 10^3^	~10^2^ (tiny^b^)	0
6	WNPPI5	Gal11P and LGF2	~1 × 10^3^	0	0
7	WNPPI8	none	~1 × 10^3^	~1 × 10^3^	~1 × 10^3^
8	WNPPI8	Gal11P and LGF2	~1 × 10^3^	0	0
9	WNPPI8	Coh_wt_ and Doc_wt_	~1 × 10^3^	0	0
10	WNPPI8	Coh_wt_ and Doc_AL_	~1 × 10^3^	~1 × 10^3^	~1 × 10^3^

^a^cfu, colony-forming unit. ^b^In comparison to the regular colony size (~1 mm) from other tests, these colonies (<0.1 mm) were barely seen by eye.

### Construction of dockerin and cohesin libraries

Structural data (PDB code, 1OHZ; Fig. 1B)^22^ revealed that the recognition mechanism of the cohesin-dockerin pair from *C. thermocellum* is mainly mediated by polar interactions^22,37^. The two highly conserved serine-threonine motifs (Ser11/Thr12 and Ser45/Thr46) in dockerin serve as key recognition codes for binding to the cohesin domain^38,39^. Due to a near perfect internal two-fold symmetry of dockerin structure (Fig. 2B), the two serine-threonine motifs interact with cohesin domains in a similar manner and only one motif interacts with cohesin at one time. Both literature data^40^ and our experimental observations showed that dockerin mutants with mutations in only one of the two motifs still recognize wild-type cohesin with no apparent decrease in affinity. However, it was reported that mutations in both motifs caused a significant reduction in affinity of the dockerin mutant toward the wild-type cohesin^40^. To further verify this notion, we generated a dockerin mutant (named as Doc_AL_), which contained four mutations, Ser11Ala, Thr12Leu, Ser45Ala, and Thr46Leu, in the serine-threonine motifs. We showed that Doc_AL_ did not have strong interaction with Coh_wt_ (entry 5; Table 1 and entry 10 Table 2).

In order to obtain dockerin mutants that abolish interaction with wild-type cohesin but can potentially recognize a cohesin mutant, we generated a dockerin mutant library in which four residues (Ser11, Thr12, Ser45, and Thr46) in the two Ser/Thr motifs were fully randomized. We also generated a cohesin mutant library in which four residues (Asn37, Asp39, Gly123, and Ala125) that could potentially affect the recognition of the Ser/Thr motif of dockerin were randomized. NNK codons (N = A, C, T, or G, K = T or G; 32 variants at nucleotide level) were used to cover all 20 amino acids at each mutation site. While mutations into certain amino acid residue(s) may affect the stability and/or expression level of the resulting mutants, such effect is in general hard to predict, and therefore is not taken into consideration in the library construction process. Since most of the amino acid residues are encoded by more than one codon, this design leverages concerns of codon bias-caused difference in protein expression and experimental challenges of constructing large mutant libraries. Both the dockerin and the cohesin library had a diversity of 1.05 × 10^6^ at the nucleotide level. The dockerin mutant library was cloned into the pTRG vector to generate pTRG-Doc_lib_ in which dockerin mutants were fused to the RNAPα protein. The cohesin mutant library was cloned into the pBT vector to generate pBT-Coh_lib_ in which cohesin mutants were fused to the λcI protein.

### Identification of an orthogonal cohesin-dockerin pair through library selections

The dockerin library was first subjected to one round of negative selection against Coh_wt_ in order to eliminate dockerin mutants that retained effective interaction with Coh_wt_. Surviving cells should contain dockerin mutants that were either non-functional or lost the ability to recognize Coh_wt_. Similarly, the cohesin library was subjected to one round of negative selection against Doc_wt_ in order to eliminate cohesin mutants that retained effective interaction with Doc_wt_. Surviving cells should contain cohesin mutants that were either non-functional or lost the ability to recognize Doc_wt_. It should also be noted that some mutants that can engage in effective interaction with Coh_wt_ or Doc_wt_ might survive the negative selection if their expression levels were too low to induce a sufficient level of URA3 expression. While such possibility exists, these mutants will likely be eliminated in the positive selection due to their low expression levels.

By using plates containing 2.5 mM FOA, the survival rate of the dockerin and cohesin mutants was estimated to be 10–20%. This number was based on the comparison between the control plate (no FOA) and the selection plate (with 2.5 mM FOA; Figure S3). We subsequently examined if we could identify cohesin mutants from the reduced cohesin library after negative selection to engage functional interaction with the aforementioned Doc_AL_ mutant. To our delight, a large number of colonies were obtained after one round of positive selection between Doc_AL_ and the reduced cohesin library. We arbitrarily picked eight colonies with different sizes for DNA sequencing analysis. Seven distinct sequences were obtained, while cohesin mutants 3 and 7 converged to the same sequence (Table S2). To estimate the effectiveness of the negative selection and to eliminate false positives (e.g., beneficial host mutations) identified in the positive selection, the pBT-Coh plasmids of the seven distinctive mutants were isolated and reintroduced into the positive selection hosts that harbored either pTRG-Doc_wt_ or pTRG-Doc_AL_. We observed that all seven mutants engaged in strong interactions with Doc_AL_ and supported cell growth in the presence of 7.5 mM 3-AT. On the other hand, six out of the seven mutants were not able to support cell growth when Doc_wt_ was co-expressed. Apparently, these cohesin mutants did not interact with Doc_wt_, which indicated that our established negative selection protocol was highly effective.

We arbitrarily picked cohesin mutant 1 (Coh_1_; Asn37Leu, Asp39Thr, Gly123Leu, and Ala125Leu; Table S2) for the subsequent selection against the reduced dockerin library. Among a few hundred survived colonies, five were picked and the corresponding pTRG-Doc plasmids were isolated and reintroduced into the positive selection hosts that harbored either pBT-Coh_wt_ or pBT-Coh_1_. Cell growth test confirmed that all five dockerin mutants engaged in strong interactions with Coh_1_ and four out of five did not interact with Coh_wt_. One dockerin mutant displayed moderate interaction with Coh_wt_. Based on the colony size and growth rate on the positive selection plate, we chose a dockerin mutant (named as Doc_1_; Ser11Arg, Thr12Pro, Ser45Pro, and Thr46Ala) for the following *in vitro* characterization.

### *In vitro* characterization

Previous reports suggested that dockerin domain could not be produced as a discrete entity due to its degradation in *Escherichia coli*^22^. On the other hand, large quantity of dockerin domain could be obtained when it was co-expressed with cohesin^22,41^. In addition, many reports showed that good expression of dockerin could be achieved when it was fused to well-folded proteins^32–35^. To this end, dockerin domain variants (including its N-terminal X6b domain) were expressed as a C-terminal fusion to the maltose binding protein (MBP). A His_6_ tag was added to the C-terminus of the fusion protein to facilitate the purification and ELISA experiments. Cohesin domain variants were purified as a C-terminal fusion to the glutathione S-transferase (GST). The GST tag improved expression of cohesin domains, facilitated protein purification, and did not interfere with the ELISA experiments using anti-His_6_ antibody. We have also verified that GST does not interact with MBP.

To estimate the strength of interaction between cohesin and dockerin, we conducted semi-quantitative ELISA experiments. Briefly, wells of microtiter plates were coated with a GST-tagged cohesin. Different concentrations of the His_6_-tagged dockerin of interest were then applied into each well. Following washing steps, the amounts of interacting dockerin were determined immunochemically using anti-His_6_ antibody and HRP-labeled secondary antibody. As shown in Table 3, Doc_1_ and Coh_1_ displayed a strong mutual interaction with a *K*_d_ value of 4.57 ± 1.61 nM, which is comparable to that of the parent Doc_wt_-Coh_wt_ pair (*K*_d_ = 0.77 ± 0.10 nM). On the other hand, the Doc_1_-Coh_1_ pair did not show obvious cross-interaction with the Doc_wt_-Coh_wt_ pair. The *K*_d_ values of the Doc_1_-Coh_wt_ and Doc_wt_-Coh_1_ cross pairs were too large to be accurately measured, which were estimated to be larger than 500 nM (Table 3 and Figure S4). The ELISA experiments confirmed that the Doc_1_-Coh_1_ pair is orthogonal to the parental Doc_wt_-Coh_wt_ pair. To verify that the evolved Doc_1_ mutant does not have increased level of non-specific interaction with other proteins due to a few hydrophilic-to-hydrophobic mutations, we conducted ELISA experiments between Doc_1_ and a control protein, BSA. The *K*_d_ value of the Doc_1_-BSA interaction was too large to be accurately measured. It is estimated to be larger than 1,500 nM, which is similar to that of the Doc_wt_-BSA pair (*K*_d_ > 1000 nM; Figure S4). We therefore concluded that mutations in Doc_1_ do not promote non-specific binding.

**Table 3:** Kd values of cohesin-dockerin pairs.

	Doc_wt_-Coh_wt_	Doc_1_-Coh_1_	Doc_wt_-Coh_1_	Doc_1_-Coh_wt_
*K*_d_ (nM)	0.77 ± 0.10	4.57 ± 1.61	>500	>500

### Conclusion

In summary, we have developed an approach to generate an interacting protein pair that is derived from but orthogonal to the parent protein pair. This is achieved by engineering the protein-protein interacting interface and facilitated by a combination of positive and negative selections in bacteria. To the best of our knowledge, our negative selection is the first example of applying URA3/5-FOA selection in a bacterial two-hybrid system. Presumably, more than one orthogonal protein pair can be generated with multiple cycles of positive and negative selections. The approach and tools that were developed in the current work can also potentially be applied to the generation of other orthogonally interacting proteins pairs of one’s interest. These orthogonal protein pairs can potentially be applied to the assembly of artificial protein complexes both *in vitro* and *in vivo*. The precise control of relative contents and positions of building blocks within a protein assembly will likely facilitate the construction of protein complexes for protein-based nanomaterials or for efficient catalytic syntheses of bio-based chemicals through co-localization of enzymes

## Methods

### Materials and General Methods

Primers were ordered from Sigma. DNA sequencing services were provided by Eurofins MWG Operon. Restriction enzymes, antarctic phosphatase (AP) and T4 DNA ligase were purchased from New England Biolabs. KOD hot start DNA polymerase was purchased from EMD Millipore. Standard molecular biology techniques^42^ were used throughout. Site-directed mutagenesis was carried out using overlapping PCR. *E. coli* XL1-Blue MRF′ was used in the construction and DNA propagation of all plasmids that were derived from pBT and pTRG vectors. *E. coli* GeneHogs were used for routine cloning and DNA propagation of all other plasmids. All solutions were prepared in deionized water that was further treated by Barnstead Nanopure® ultrapure water purification system (Thermo Fisher Scientific Inc). LB medium (1 L) contained Bacto tryptone (10 g), Bacto yeast extract (5 g), and NaCl (10 g). M9 salts (1 L) contained Na_2_HPO_4_ (6 g), KH_2_PO_4_ (3 g), NH_4_Cl (1 g), and NaCl (0.5 g). M9 glucose medium contained glucose (10 g), MgSO_4_ (0.12 g), CaCl_2_ (0.028 g) and thiamine hydrochloride (0.001 g) in 1 L of M9 salts. Antibiotics were added where appropriate to following final concentrations: ampicillin, 100 mg L^−1^; kanamycin, 50 mg L^−1^; chloramphenicol, 25 mg L^−1^; tetracycline, 12.5 mg L^−1^. The 6xHis tag monoclonal antibody was purchased from Thermo Fisher Scientific. The goat anti-mouse IgG-HRP conjugate was purchased from BioRad.

### Plasmid construction

Plasmid pBT-Coh_wt_ was constructed by inserting Coh_wt_-encoding gene (the second cohesin domain from CipA; residues 182–328) between the *EcoR*I and *Bam*HI sites of the pBT vector. Coh_wt_-encoding gene was PCR amplified using primers P1 and P2 (Table S1) from the chromosomal DNA of ATCC 27405.

Plasmid pTRG-Doc_wt_ was constructed by inserting Doc_wt_-encoding gene (the dockerin domain from xylanase 10B; residues 733–791) between the *Bam*HI and *Xho*I sites of the pTRG vector. Doc_wt_-encoding gene was PCR amplified using primers P3 and P4 (Table S1) from the chromosomal DNA of ATCC 27405. DNA sequence that encodes X6b carbohydrate binding domain was included at the 5′ end of the Doc_wt_-encoding gene.

Plasmid pTRG-Doc_AL_ was constructed by site-directed mutagenesis of plasmid pTRG-Doc_wt_. Mutations (Ser11Ala, Thr12Leu, Ser45Ala, and Thr46Leu) were introduced by overlapping PCR using primers P5, P6, P7, P8, and P9 (Table S1). The resulting DNA fragment was inserted into the pTRG vector using Ligation Independent Cloning (SLIC)^43^.

Plasmids pGEX-Coh_wt_ and pGEX-Coh_1_ were constructed by inserting Coh_wt_-encoding gene and Coh_1_-encoding gene between the *EcoR*I and *Xho*I sites of pGEX vector, respectively. As a result, the cohesin variants were expressed as fusion protein to the C-terminus of GST. The Coh_wt_-encoding gene and Coh_1_-encoding gene were amplified using primers P10 and P11 (Table S1).

Plasmids pMAL-Doc_wt_, pMAL-Doc_AL_, and pMAL-Doc_1_ were constructed by inserting a dockerin gene of interest between the *Bam*HI and *Xho*I sites of pMAL vector, respectively. The dockerin variants were expressed as C-terminus fusion of MBP. The dockerin encoding genes were amplified using primers P3 and P12 (Table S1).

### Construction of a dockerin library

Four residues (Ser11, Thr12, Ser45, and Thr46) of dockerin were randomized. Primers P29 and P30 (Table S1) were used to introduce these mutations. The full-length dockerin mutant fragments were assembled by overlapping PCR of the above two DNA fragments using primers P3 and P4 (Table S1). The resulting DNA was digested with *Bam*HI and *Xho*I, and subsequently cloned into the pTRG vector that was digested with the same pair of restriction enzymes to produce the dockerin library.

### Construction of a cohesin library

Four residues (Asn37, Asp39, Gly123, and Ala125) of cohesin that are in contact with the Ser/Thr motif of dockerin were randomized. Primers P13 and P14 (Table S1) were used to amplify a DNA fragment that contains mutations at positions Gly123 and Ala125. Primers P15 and P16 were used to amplify a DNA fragment that contains mutation at positions Asn37 and Asp39. The full-length cohesin mutant fragments were assembled by overlapping PCR of the above two DNA fragments using primers P1 and P2 (Table S1). The resulting DNA was digested with *EcoR*I and *Bam*HI, and subsequently cloned into the pBT vector that was digested with the same pair of restriction enzymes to produce the cohesin library.

### Construction of host strain for negative selection

Chromosomal deletion and modification of the F’ plasmid was carried out using the phage λ Red-mediated homologous recombination^44^. In brief, an appropriate *E. coli* host strain was first transformed with plasmid pRed-ET (Gene Bridges). A single colony of transformed cells was cultured in LB medium containing Ap at 30 °C. Expression of the λ Red recombination proteins was induced with L-arabinose at a final concentration of 0.4% (wt/vol), when the cell growth reached the mid exponential phase. The cultivation temperature was shifted to 37 °C. Following additional 1 h of growth, the cells were harvested for the preparation of recombination-ready electrocompetent cells. To knockout the chromosomal *pyrF* gene (encodes orotidine-5′-phosphate decarboxylase), linear DNA fragment was assembled by overlapping PCR using primers P17, P18, P19, P20, P21, and P22. The DNA contains a chloramphenicol acetyltransferase-encoding gene flanked by DNA sequences (50–60 bp) that are homologous to the upstream and downstream regions of the *pyrF* gene. Following transformation into the BacterioMatch II reporter strain, successful gene deletion event was selected by plating cells on solid media containing chloramphenicol. The chloramphenicol acetyltransferase selection marker was subsequently removed from the genome using FLP recombinase^44^ to yield strain WNPPI7, which is a uridine auxotroph. Insertion of the URA3 gene behind the HIS3 reporter gene on the F’ plasmid of WNPPI7 was achieved by electroporation of DNA fragment that was constructed by overlapping PCR using primers P23, P24, P25, P26, P27, and P28. Successful gene insertion event led to the recovery of the uridine auxotrophic phenotype. Strains grown on M9 media containing glucose as the sole carbon source were evaluated and characterized to result in WNPPI8.

### Negative selection

The negative selections were performed to eliminate the cohesin and the dockerin mutants that could interact with Doc_wt_ and Coh_wt_, respectively. For negative selection with the cohesin library, WNPPI8 cells containing pBT-Coh_lib_ and pTRG-Doc_wt_ were cultivated in 5 mL LB medium overnight. The overnight culture (1 mL) was collected using a tabletop centrifuge at 2000 *g* for 10 minute at room temperature. After removal of the LB medium, cells were washed once using 1 mL of M9 complete medium (1 × M9 salts, 1 mM MgSO_4_, 0.1 mM CaCl_2_, 10 mg/L thiamine, 0.01 mM ZnSO_4_, 0.2 mM uracil, 1 g/L histidine, 0.01% yeast extract, 0.4% D-glucose), then re-suspended in 1 mL of the same medium. The cells were incubated at 37 °C for 2 hours with shaking (225 rpm), which allowed cells to adapt to the growth in minimal medium before plating. Around 5 × 10^6^ cells were plated on M9 complete medium plates containing 2.5 mM 5-FOA, 0.05 mM IPTG, and appropriate concentrations of chloramphenicol and tetracycline. After 36 h of incubation at 37 °C, cells were collected from the plate into 1 mL M9 complete medium. Plasmid DNA was extracted from the collected cells. Mixture of the pBT-Coh_lib_ and pTRG-Doc_wt_ plasmids was first treated by restriction enzyme to linearize the pTRG-Doc_wt_ plasmid. The pBT-Coh_lib_ plasmids were then isolated by DNA gel electrophoresis purification. Negative selection with the dockerin library was conducted using the same procedure.

### Positive selection

For positive selections with the reduced cohesin library, the pBT-Coh_lib_ from the negative selection was transformed into the BacterioMatch II reporter strain containing a pTRG-Doc variant of interest. The positive selection followed instructions of the BacterioMatch II two-hybrid kit. In brief, overnight bacterial culture in LB medium (1 mL) was collected by centrifugation. Cell pellets were washed with M9-His drop medium (1 × M9 salts, 1 mM MgSO_4_, 0.1 mM CaCl_2_, 10 mg/L thiamine, 0.001 mM ZnSO_4_, 1 × His-drop supplement, 0.2 mM adenine, 0.4% D-glucose) to completely remove the residue LB medium. Cells were then re-suspended in 1 mL M9-His drop medium and incubated for 2 h at 37 °C with shaking (225 rpm). An aliquot of cells (3 × 10^6^) was plated on M9-His drop medium plates containing 5 mM 3-AT, 0.05 mM IPTG, and appropriate concentrations of chloramphenicol and tetracycline. Positive selections with the reduced dockerin library flowed the same procedure.

### Enzyme-linked immunosorbent assay (ELISA)

MaxiSorp 96 well ELISA plates were coated overnight at 4 °C with 100 μL of the desired protein (30 nM in 0.1 M Na_2_CO_3_ (pH 9.6)). Following steps were all performed at a volume of 100 μL/well at room temperature unless otherwise stated. After removal of the coating solution, blocking buffer (1 mM CaCl_2_ and 1% BSA in TBS buffer) was added and the plates were incubated for 1 h. The plates were washed three times with washing buffer (blocking buffer supplemented with 0.05% Tween 20 without BSA; 200 μL/well per wash). Potential binding partner of the coating protein (10 pM–1 μM in blocking buffer) was applied to each well. The plates were again incubated for 1 h followed by washing for three times. Primary antibody (anti-6xHis, 1:500 dilution in blocking buffer) was added and incubated for 1 h. Plates were washed. Secondary antibody (goat anti-mouse IgG-HRP conjugate, 1:500 dilution in blocking buffer) was added for the final incubation of 1 h followed by washing for three times. Quantification of the HRP activity used the 3, 3′, 5, 5′-tetramethylbenzidine (TMB) substrate. The reaction was initiated by the addition of 100 μL/well TMB. After 5 min of reaction or until the desired color development achieved, 50 μL/well of 1 M H_2_SO_4_ was added to terminate the reaction. Absorbance at 450 nm was measured. Dissociation constants were calculated by curve fitting with Hill Equation using MATLAB (R2016b).

## Electronic supplementary material

Supplementary information
