# Supplementary material for: A high throughput approach for the generation of orthogonally interacting protein pairs
Source: Sci Rep. 2018 Jan 17;8:867. doi: 10.1038/s41598-018-19281-6 (PMC5772552; doi:10.1038/s41598-018-19281-6)
Supplement: Supplementary file 1 — Supplementary information [file 41598_2018_19281_MOESM1_ESM.pdf]

A high throughput approach for the generation of orthogonally interacting protein pairs

Justin Lawrie,[1+] Xi Song,[1+] Wei Niu,[2*] Jiantao Guo[1*]

1. Department of Chemistry, University of Nebraska-Lincoln, Lincoln, Nebraska, 68588, United States.

2. Department of Chemical & Biomolecular Engineering, University of Nebraska-Lincoln, Lincoln, Nebraska, 68588, United States.

*To whom correspondence should be addressed: jguo4@unl.edu and wniu2@unl.edu

[+]These authors contributed equally to this work.

Supporting Information

Table of Contents

I. Table S1: 3

II. Table S2 4

III. Figure S1.......... 5

IV. Figure S2..........6

V. Figure S3.......... 7

VI. Figure S4..........8

Table S1. The list of primers for plasmid and library constructions.

name	sequence
P1	5'- GTCAGAATTCAGACGGTGTGGTAGTAGAAATTGGCAAAG-3'
P2	5'- CAGTGGATCCTTACTTGGTCGGTGTTGCATTGCCAACG-3'
P3	5'-GAGGATCCAACGGATATTATTATCATGACACTTTTG-3'
P4	5'-GACTCGAGTTAGATTACTCTTAAAAGGTAGCGTG-3'
P5	5'-TCCGCAATACAGAATTCCTTC-3'
P6	5'-GTTAATGGTTCCATCACCGTTTAC-3'
P7	5'-GATGGAACCATTAACGCACTGGACTTGACAATGTTAAAGAG-3'
P8	5'-TGAAAGAAGTAAAACATCCAGCGCGTTTATCGATCCATTCTTGTC-3'
P9	5'-TAATTAATTACTCGAGTTAGATTACTCTTAAAAGGTAGCGTGAAAGAAGTAAAAC-3'
P10	5'-GTCAGAATTCGACGGTGTGGTAGTAGAAATTGGCAAAG-3'
P11	5'-CTAGCTCGAGTTACTTGGTCGGTGTTGCATTGCCA-3'
P12	5'-GACTCGAGGATTACTCTTAAAAGGTAGCGTG-3'
P13	5'-ACTTTCGACGAAGTAGGTNNKTTTNNKGATAATGACCTGGTAGA-3'
P14	5'-ACCTACTTCGTCGAAAGT-3'
P15	5'-GAGTTCCATCCAAAGGAATAGCANNKTGCNNKTTTGTGTTCAGATATGATCCGAATG-3'
P16	5'-TATTGCAGTATCAAAGCTCTTGG-3'
P17	5'-TGCTGATATCATCGAAGCGCGCGAC-3'
P18	5'-AGCCTACACGACCAGACCTTCTTGATGATGGGCAC-3'
P19	5'-GTCTGGTCGTGTAGGCTGGAGCTGCTTCGAAG-3'
P20	5'-GAATCACTCACATATGAATATCCTCCTTAGTTCCTATTCCGAAG-3'
P21	5'-TTCATATGTGAGTGATTCCAACAGCCGTCTGGTC-3'
P22	5'-AGGTGGTGCAGCTGTCGGAGGTG-3'
P23	5'-GACCATCACACCACTGAAGACTGC-3'
P24	5'-AAACAGCTATGTCGAAAGCTACATATAAGGAAC-3'
P25	5'-TACAGCGCATTTACTTATAATACAGTTTTTAGTTTTG-3'
P26	5'-AAGTAAATGCGCTGTAGAAGTCACCATTG-3'
P27	5'-TTCGACATAGCTGTTTCCTGTGTGGGATC-3'
P28	5'-GACTACCTTGGTGATCTCGCCTTTCAC-3'
P29	5'-GTGATGGAACCATTAACNNKNNKGACTTGACAATGTTAAAG-3'
P30	5'-GACAAGAATGGATCGATAAACNNKNNKGATGTTTTACTTCTTTCACG-3'

Table S2. Mutations in the obtained cohesin variants.

Coh_{wt}	Asn37	Asp39	Gly123	Ala125
Coh_1	Leu	Thr	Leu	Leu
Coh_2	Lys	Phe	Trp	Val
Coh_3	Ala	Val	Val	Trp
Coh_4	Val	Ala	Leu	Leu
Coh_5	Cys	Asn	Val	Gly
Coh_6	Val	Thr	Trp	Ile
Coh_7	Ala	Val	Val	Trp
Coh_8	Ser	Val	Val	Trp

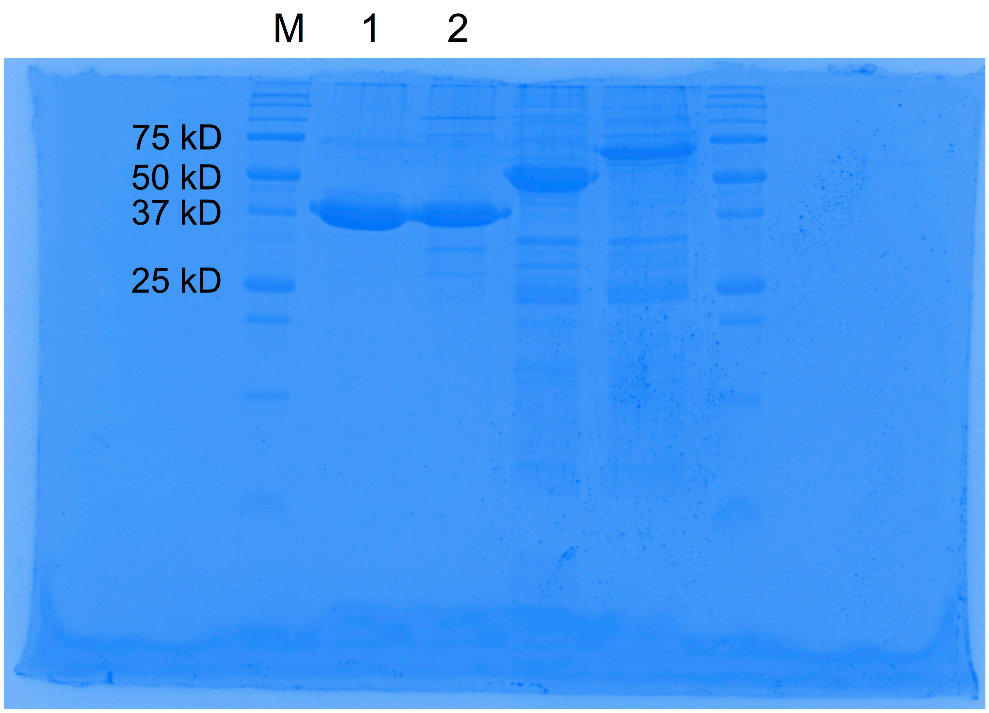

Figure S1. SDS-PAGE analysis of purified cohesin variants. Lane M, molecular weight marker; Lane 1, GST-Coh_{wt}; Lane 2, GST-Coh_1.

Note: Protein bands in other lanes are not for this work.

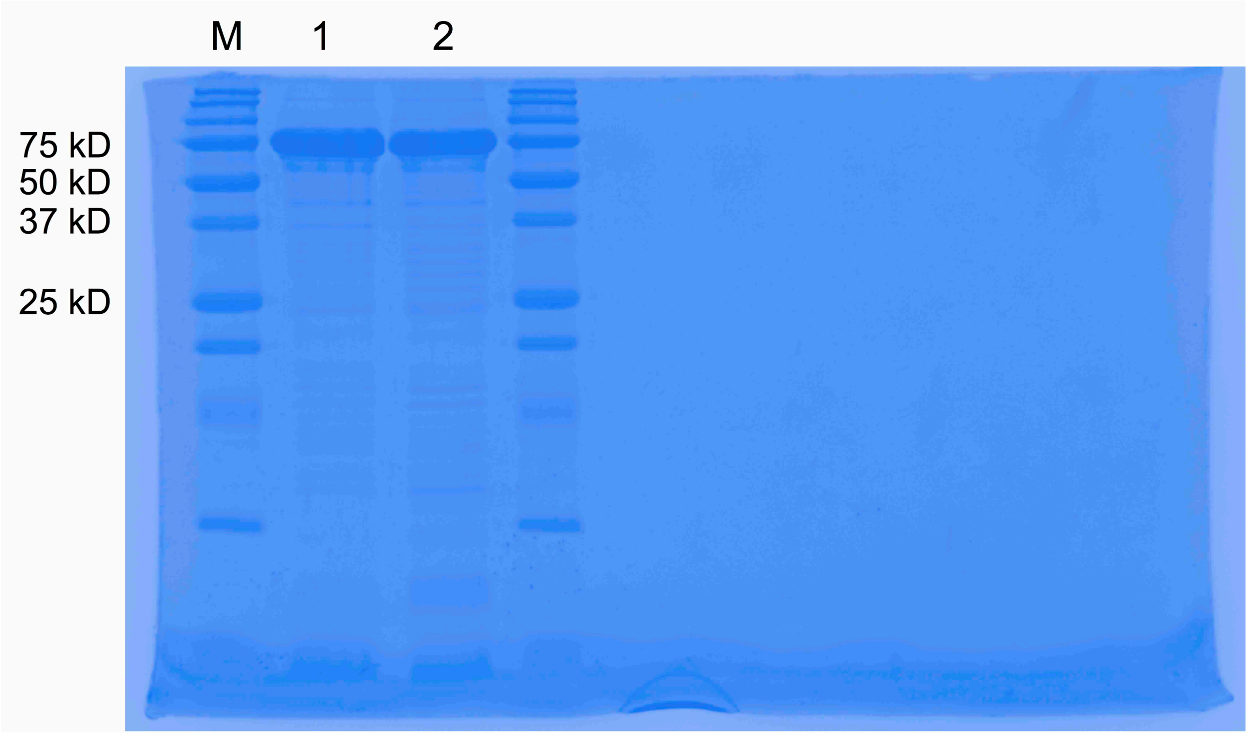

Figure S2. SDS-PAGE analysis of purified dockerin variants. Lane M, molecular weight marker; Lane 1, MBP-Doc_{wt}; Lane 2, MBP-Doc_1.

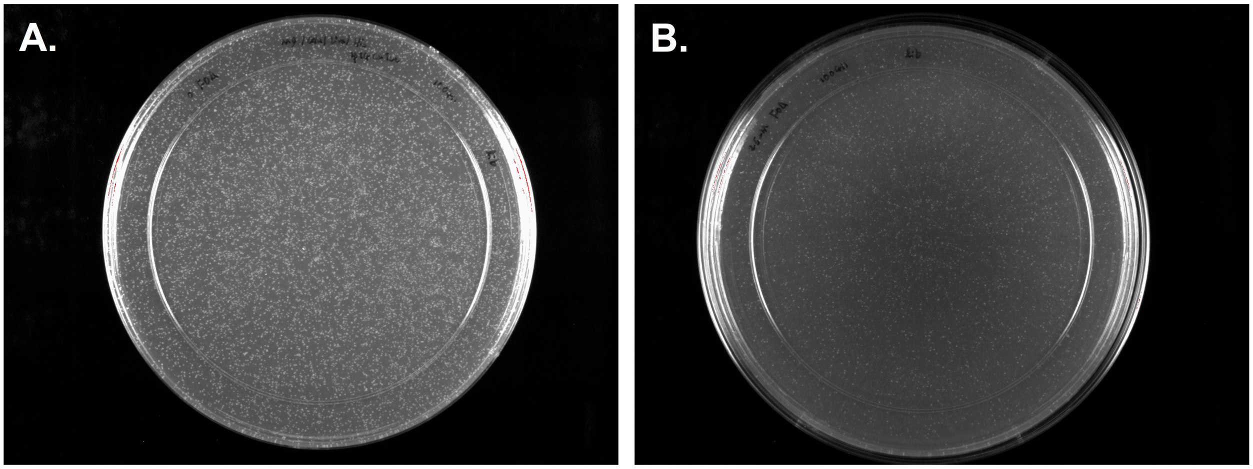

Figure S3. A typical negative selection between the dockerin (or cohesin) library and the wild-type cohesin (or dockerin). (A) Library selection in the absence of 5-FOA; (B) Library selection in the presence of 2.5 mM 5-FOA. Based on an eye examination of colony-forming units in the absence and in the presence (2.5 mM) of 5-FOA, the survival rate of the mutant library after negative selection was estimated as 10-20%.

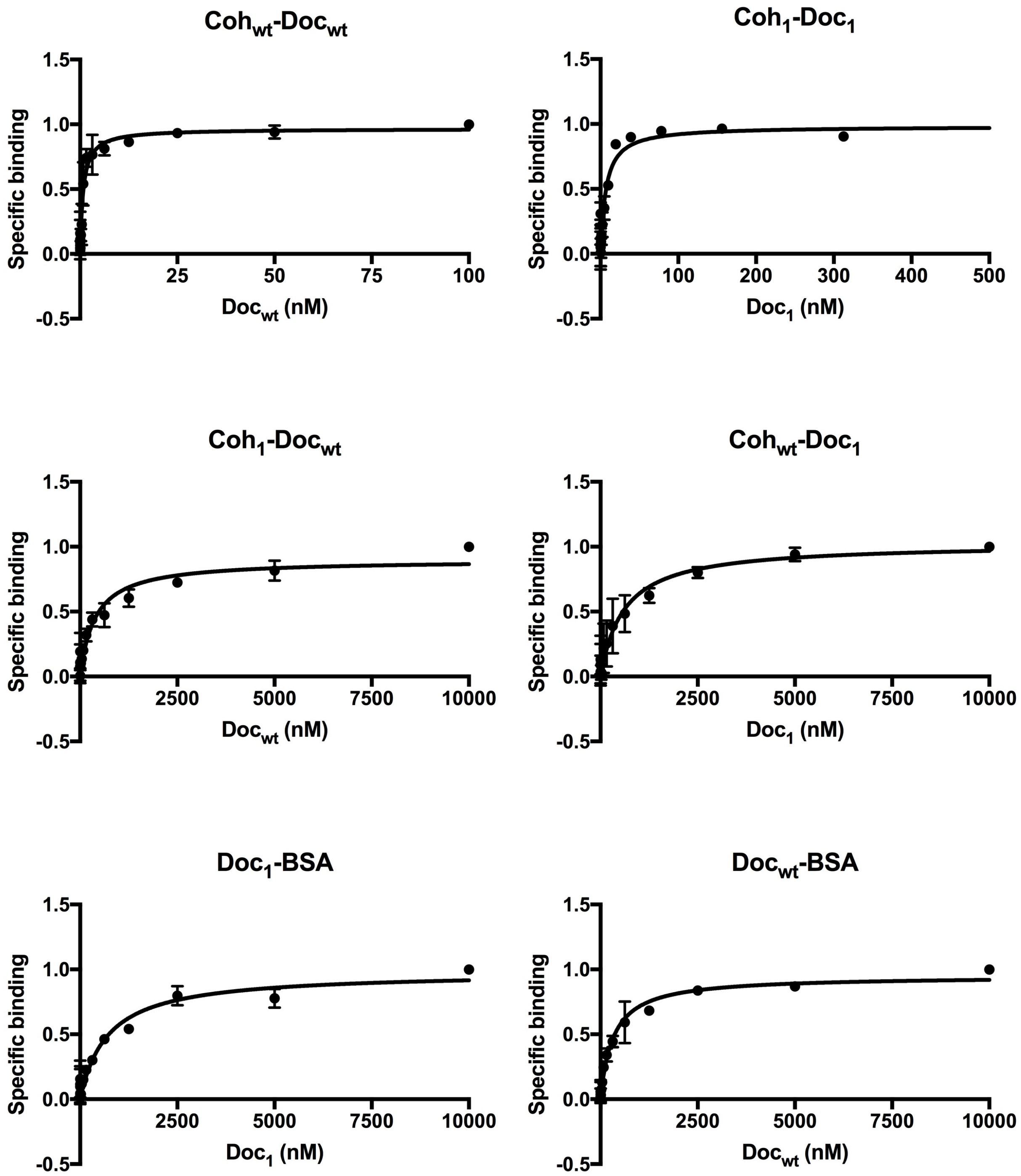

Figure S4. Binding curves obtained from ELISA experiments.
